# Cryotolerance of apple tree bud is independent of endodormancy

**DOI:** 10.3389/fpls.2015.00695

**Published:** 2015-09-08

**Authors:** Alois Bilavcik, Jiri Zamecnik, Milos Faltus

**Affiliations:** Plant Physiology and Cryobiology Laboratory, Crop Research InstitutePrague, Czech Republic

**Keywords:** dormancy, endodormancy, vegetative bud, apple (*Malus domestica* Borkh.), low-temperature survival, cryopreservation

## Abstract

Increasing interest in cryopreservation of dormant buds reveals the need for better understanding of the role of dormancy in cryotolerance. Dormancy stage and low-temperature survival of vegetative apple buds (*Malus domestica* Borkh.), cultivars ‘Sampion’ and ‘Spartan’, collected from orchard were evaluated during three seasons contrasting in temperature and precipitation throughout the arrested plant growth period. During each season, the cultivars differed either in the onset of the endodormancy or in the length of the endodormant period. A simple relation between endodormancy of the buds and their water content was not detected. The cryosurvival of vegetative apple buds of both cultivars correlated with their cold hardening without direct regard to their particular phase of dormancy. The period of the highest bud cryotolerance after low-temperature exposure overlapped with the endodormant period in some evaluated seasons. Both cultivars had the highest cryosurvival in December and January. The presented data were compared with our previous results from a dormancy study of *in vitro* apple culture. Endodormancy coincided with the period of successful cryosurvival of apple buds after liquid nitrogen exposure, but as such, it was not decisive for their survival and did not limit their successful cryopreservation.

## Introduction

Plants in temperate and arctic zones are annually exposed to periodic changes in external conditions, especially temperature, precipitation, and day length. During their phylogenetic evolution, plants adapted to these changes by creating regular growth cycles, which are synchronized with the change of environment during the different seasons. In these cycles, growth activity and dormancy alternate. However, dormancy is a state permitting plants to survive unfavorable periods; it is considered in this context as adaptation of plants to climatic cycle and not as a response to adverse climatic conditions (Lang et al., 1987). Each plant species developed its own strategy to survive adverse conditions, and, therefore, it is difficult to find a simple explanation of a universal principle mechanism for managing the process of dormancy (Dennis, 1994). Buds of trees retain their growth and encapsulate by scales. Within deciduous trees, the entrance into dormancy is accompanied by leaf fall; trees are becoming resistant to drought and other adverse conditions. According to the ability of dormant buds to grow at favorable conditions, Lang et al. (1987) divide dormancy in three phases. The first phase of dormancy, paradormancy (also known as correlative inhibition), occurs when the growth is hampered by external physiological factors of the affected bud, but within the plant, there is typically involved the influence of one organ over another one, e.g., apical dominancy. The next phase, endodormancy (also called as true dormancy), is characterized by inability of bud growth in favorable conditions. In the endodormant phase, the growth is arrested by internal physiological factors. The following ecodormancy is a phase when growth is limited by external environmental factors such as temperature extremes or lack of water. The length of endodormant phase is influenced by several factors as by daylight and water availability, but the main factor is a course of low temperatures (Crabbe and Barnola, 1996). The length of dormancy also depends on the genotype (Palonen and Linden, 1999). Endodormancy can be induced even in *in vitro* cultures of apples by their exposure to cold acclimation conditions (Bilavcik et al., 2012).

In winter, the unfavorable part of the year for plant growth, dormancy of trees of mild and cold climates is associated with increased frost hardiness. Endodormancy is physiologically the most important part of dormancy in winter (Faust et al., 1991). During endodormancy, most water in bud meristem, floral primordia, bud base, and whole bud becomes bound and unfreezable, which also enhances the frost tolerance of plants grown in temperate zone (Faust et al., 1991; Buban and Faust, 1995; Erez et al., 1998).

Cryopreservation is a method for preservation of biological material. Cryopreservation of apple germplasm belongs to one of the important methods of preservation genetic resources of plant material. Cryopreservation can act as a safe duplicate of field or *in vitro* collections.

In general, cryopreservation of dormant buds, two step cryopreservation, is based on removing excessive water by freeze-induced dehydration in the first step – slow cooling down to temperatures close to –30°C and following immersion into liquid nitrogen (LN) as the second step. The success of this method is given by sampling the buds in a defined physiological stage of development when they are at maximum of cold hardiness or when they can induce their hardiness in controlled conditions. *In vitro* apple cryopreservation technique is based on removing excessive free water, causing freezing injury, from meristematic tissues either by dehydration in the air or by application of cryoprotective solutions (Niino and Sakai, 1992; Wu et al., 1999; Sedlak et al., 2001). Although, the cryopreservation of *in vitro* cultures has an advantage in the availability of uniform plant material throughout the whole year, the cryopreservation of dormant buds has taken place in the last decade due to a less time-demanding procedure and a development of methods with high reproducibility.

Sakai (1960) has given a background for cryopreservation of dormant buds of many trees using a method by which the dormant-winter twigs of *Salix* and *Populus* survived the LN temperature after slow pre-freezing to –30°C. The effective cryoprotocol for apple dormant buds was developed at Fort Collins, CO, USA (Tyler and Stushnoff, 1988b; Stushnoff and Seufferheld, 1995; Forsline et al., 1998; Towill et al., 2004; Towill and Ellis, 2008; Jenderek et al., 2011). The dormant bud cryoprotocol was developed and implemented on large scale in regions with a continental climate with relatively hard winters. In Europe, cryopreservation of apple dormant buds was done from the North, e.g., Finland – *Salix* (Ryynanen, 1996) to the South, e.g., Italy (Lambardi, 2012) from the West, e.g., Denmark (Toldam-Andersen et al., 2007; Vogiatzi et al., 2011) to the East, e.g., Germany (Hofer, 2007, 2015), and The Czech Republic (Zamecnik et al., 2007). Dormant bud cryopreservation started to develop also in Kazachstan (Kovalchuk et al., 2014) and other Asian regions. In all these cryopreservation centers on different continents with different winter climates, the donor trees of buds for cryopreservation are supposed to be in a dormant state (they do not distinguish different parts of dormancy) even though their cryopreservation results vary with season. Although, the dormant state of the buds subjected to cryopreservation is a prerequisite (Stushnoff, 1987; Tyler and Stushnoff, 1988b), the effect of a true dormancy stage on cryotolerance, the endodormancy, has not been studied yet. Due to increasing interest in cryopreservation of dormant buds, we reevaluated the most appropriate ecological data from three following seasons with extreme ecological conditions to reveal the role of endodormancy in cryosurvival. The aim of our work was to test the hypothesis that endodormancy requirement is necessary for successful survival of apple dormant buds after cryopreservation at temperatures of LN.

## Materials and Methods

**FIGURE 1:**
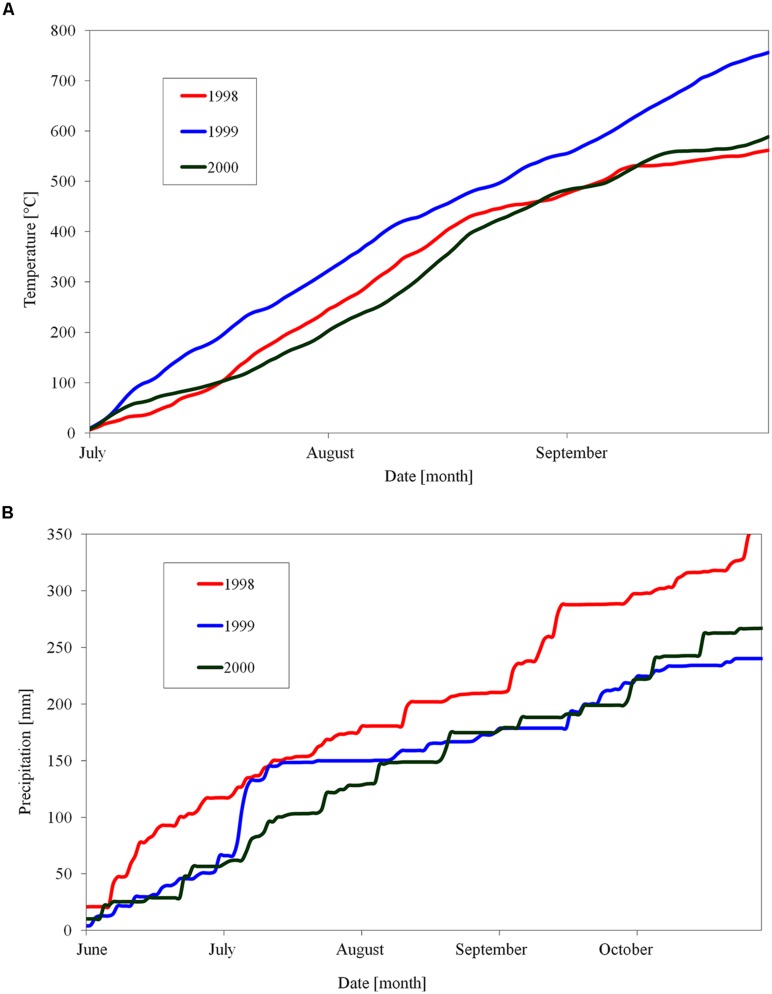
**Cumulative sum of day temperatures above 10°C **(A)** and day precipitation **(B)** in orchard of Crop Research Institute, Prague, Czech Republic, during three seasons of 1998, 1999, and 2000**.

One-year shoots of apple (*Malus domestica* Borkh.) cultivar ‘Sampion’ and ‘Spartan’ were taken from trees in an orchard of Crop Research Institute, Prague, during three winter seasons of 1998/1999, 1999/2000, and 2000/2001. Both cultivars were grown on M4 rootstocks. The apple trees of cultivar ‘Sampion’ and ‘Spartan’ were 16 and 7 years old, respectively. The cultivars were selected because of a good resistance to low temperatures. The orchard location is characterized by altitude 350 m. a. s. l., 7.9°C average annual temperature, 394 mm average annual precipitation, and the average day length from August to November from 14.5 h to 9 h 5 min, respectively. The temperature and precipitation for a period of time before sampling the shoots is shown in **Figure 1**. One-year shoots were sampled from the treetop between 9 am and 11 am in 1-week intervals from September to March. The middle part of shoots was cut into one-nodal segments. Water content was evaluated in a set of three randomly sampled one-nodal segments per variant. They were weighed immediately after cutting, and then put into dryer and dried out at the temperature 105°C for at least 48 h into constant weight. The water content was counted as a difference between fresh and dry weight and expressed gravimetrically in gH_2_O g^-1^dry matter (DM).

### Endodormancy Measurement

One-year shoots were taken once a week from the orchard during September to March. Leaves from the shoots were removed if present. The shoots were cut into one-nodal segments. The segments from the upper and lower part of the shoot were excluded. Three sets of 15 segments were placed into moist peat at 20°C, 75% relative humidity (RH), 16/8 (light/dark) photoperiod and 110 μE s^-1^ m^-2^ PAR. Growth of buds from segments was evaluated after 30 days. When the bud sprouted more than 5 mm it was marked as growing bud. All non-sprouted buds were longitudinally cut and brown/necrotic buds were excluded from the evaluation set. If less than 50% of buds sprouted the buds in the sampling date were considered endodormant.

### Cryopreservation Determination

One-year-old shoots of both cultivars, ‘Sampion’ and ‘Spartan’, were cut into 2–2.5 mm long one-nodal segments with the bud in a central position. Only segments from the middle part of the shoots were used. The dormant buds were subjected to cryopreservation procedure in two variants. The first variant, non-dehydrated (ND) variant, was cryopreserved immediately after sampling from the orchard. The second variant, dehydrated (D) variant, was cryopreserved after frost dehydration of at least 1 week at –4°C in walk in chamber. In each variant, there were two sets. The first set of segments was used for evaluation of survival after 24 h at –30°C (–30), the first step of the cryoprotocol. The second set of segments was used for evaluation of survival at –196°C (LN), the second step of the cryoprotocol. Each set had three repetitions of 15 buds and the average value with SD was calculated. ANOVA was performed with STATISTICA 6.1 StatSoft Inc. (α = 0.05). Two or three segments for cryopreservation were placed into 5-ml cryovials. The cryovials were inserted in a metal holder, which was placed in an aluminum tube for cooling. The tubes were put in a plastic bag and placed in a programmable cooling ethanol bath (Ultra Kryomat Lauda RUK 50), and cooled at 2°C h^-1^ to –30°C. After 24 h at –30°C, the first set of segments was warmed up, and the second set of segments was plunged in LN and transferred into a Dewar flask. After at least 24 h in LN, the second set was warmed up. Warming was done by placing the tubes at +4°C in refrigerator for 24 h. Then, the segments were placed on a moisten filter paper in Petri dish and sealed with a foil. The Petri dish was maintained at 4°C. After 48 h, the evaluation of segment survival was done by examination of oxidative browning of tissues on the longitudinal cut of the bud under the binocular microscope (Seufferheld et al., 1999). The 9-point scale was used for evaluation. Visually intact green bud was marked as 9; bud with some browning in tissues as 5; and bud with totally brown tissues was marked as 1. Only buds marked as 9 were evaluated as survived.

## Results

### Endodormancy Time course

**FIGURE 2:**
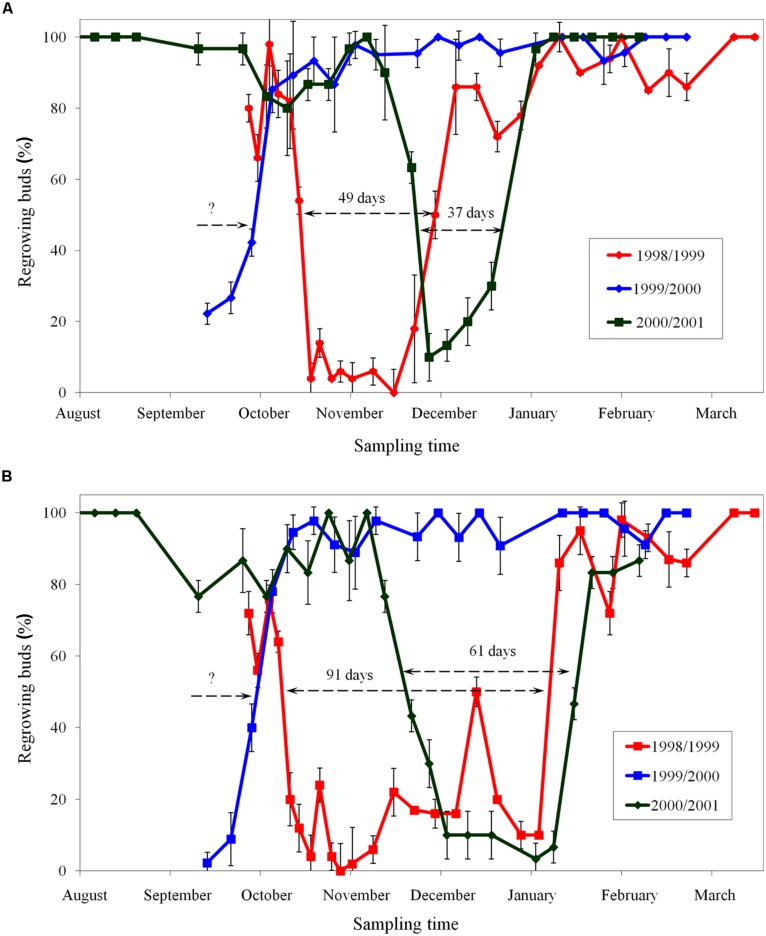
**Period of endodormancy of vegetative buds of apple cultivars ‘Sampion’ **(A)** and ‘Spartan’ **(B)** in the seasons of 1998/1999, 1999/2000, and 2000/2001**. Endodormancy was assessed when less than 50% of buds did not regrow after 30 days in growth inductive conditions. Bars indicate SD (*p* < 0.05). Note: Different beginning and the end of endodormancy. The length of arrows corresponds to the length of endodormancy (in days). The question mark indicates unknown length of endodormancy in the season 1999/2000.

The period of endodormancy of apple cultivar ‘Sampion’ and ‘Spartan’ in winter season of 1998/1999 was from October 19, 1998 to December 7, 1998 (49 days) and from October 12, 1998 to January 11, 1999 (91 days), respectively. In winter season of 1999/2000, the entering in endodormancy was not measured. The release of endodormancy of both apple cultivars ‘Sampion’ and ‘Spartan’ in winter season of 1999/2000 was October 6, 1999. The period of endodormancy of apple cultivar ‘Sampion’ and ‘Spartan’ in winter season of 2000/2001 was from November 28, 2000 to January 3, 2001 (37 days) and from November 22, 2000 to January 22, 2001 (61 days), respectively. The time course of endodormancy of apple cultivar ‘Sampion’ and ‘Spartan’ in winter seasons of 1998/1999, 1999/2000, and 2000/2001 is shown in **Figure 2**.

**FIGURE 3:**
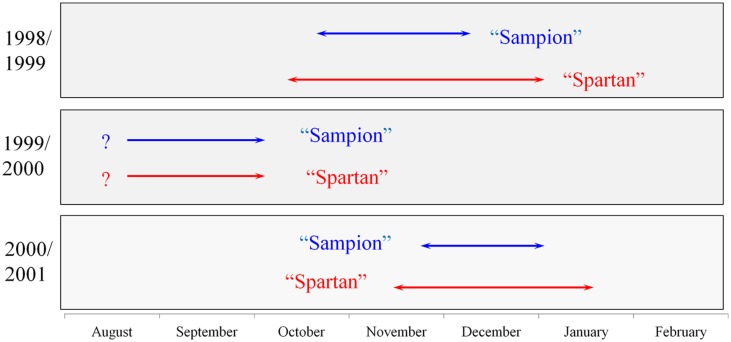
**Scheme of endodormancy of vegetative buds of apple cultivars ‘Sampion’ and ‘Spartan’ in the seasons of 1998/1999, 1999/2000, and 2000/2001.** The length of arrows corresponds to the length of endodormancy. The question mark indicates unknown length of endodormancy in the season of 1999/2000.

There were differences in the length of endodormancy among seasons, differences in time of entering and release from endodormancy among seasons, and the cultivars differed also in one season to each other (**Figure 3**). The three evaluated seasons differed in their temperature and precipitation conditions important for inducing dormancy (**Table 1**). The exact course of the temperature and precipitation is shown in **Figure 1**.

**Table 1:** Sum of daily temperatures above 10°C (from July to September) and precipitation (from June to October) in seasons of 1998, 1999, and 2000 in Crop Research Institute in Prague, Czech Republic.

Season	Sum of temperatures above 10°C (July–September) (°C)	Sum of precipitation (June– October) (mm)
1998	562	358
1999	756	240
2000	589	267

### Endodormancy and Cryopreservation

#### Winter Season of 1998/1999

The survival of ‘Sampion’ and’ Spartan’ ND variant after the first step of cryopreservation protocol (ND-30) ranged from 17 to 97% and 0 to 37%, respectively. The survival of ‘Sampion’ and ‘Spartan’ ND variant after the second step of cryopreservation protocol (NDLN) ranged from 7 to 77% and from 0 to 14%, respectively. Water content of ‘Sampion’ and ‘Spartan’ dormant buds ranged from 0.59 to 1.21 gH_2_O g^-1^DM and from 0.58 to 1.12 gH_2_O g^-1^DM, respectively.

**FIGURE 4:**
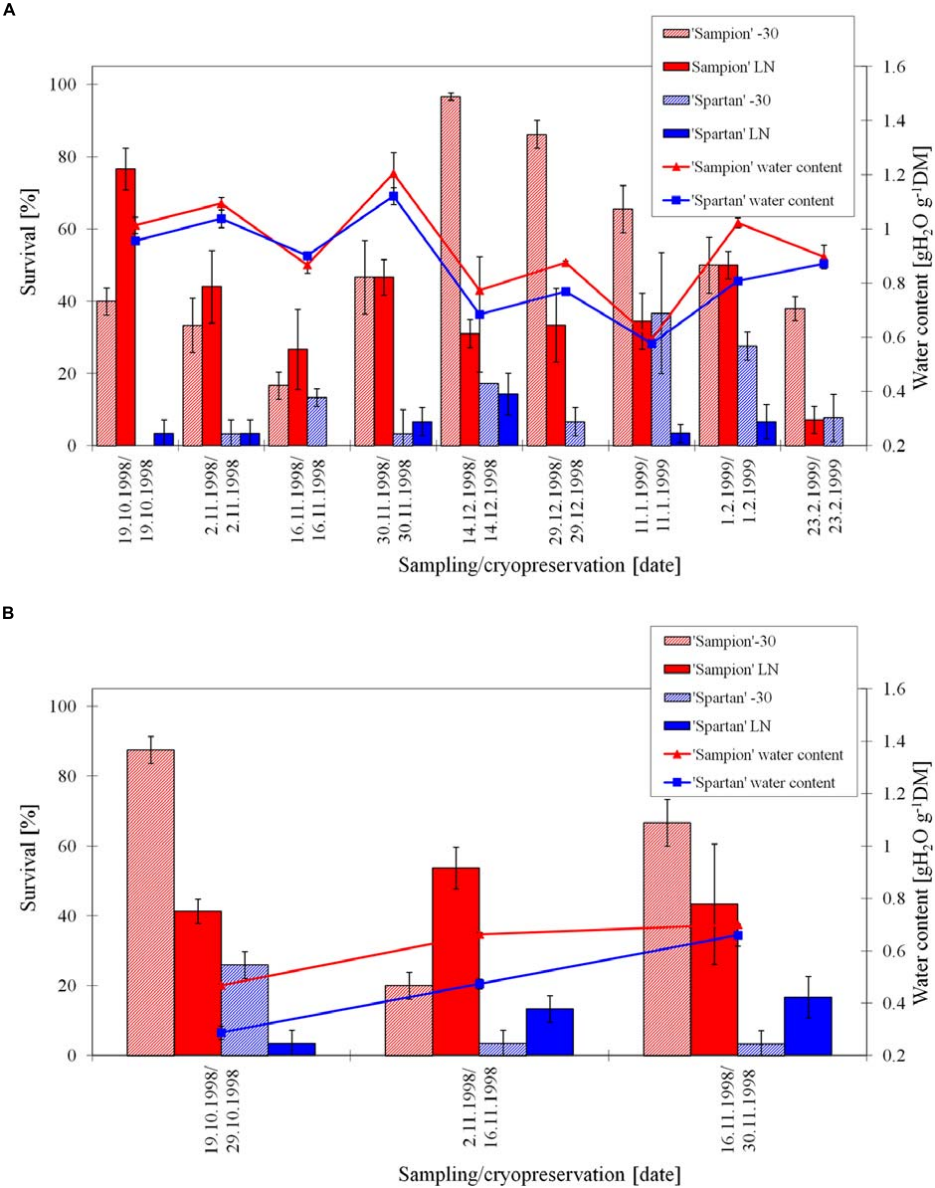
**Survival and water content of non-dehydrated **(A)** and dehydrated **(B)** vegetative buds of apple cultivars ‘Sampion’ and ‘Spartan’ **(B)** in the seasons of 1998/1999.** Non-dehydrated (ND) variant was cryopreserved immediately after sampling from the orchard. Dehydrated (D) variant was cryopreserved after frost dehydration of at least 1 week at –4°C. Survival was evaluated after the first step of cryopreservation protocol at –30°C (–30), and after the second step, reaching the liquid nitrogen (LN) temperature. Survival was tested by examination of oxidative browning of tissues on the longitudinal cut of the bud. Water content was measured immediately before cryopreservation procedure. Bars indicate SD (*p* < 0.05).

The survival of ‘Sampion’ and ‘Spartan’ D variant after the first step of cryopreservation protocol (D-30) ranged from 20 to 88% and from 3 to 26%, respectively. The survival of ‘Sampion’ and ‘Spartan’ D variant after the second step of cryopreservation protocol (DLN) ranged from 41 to 54% and from 4 to 17%, respectively. Water content of ‘Sampion’ and ‘Spartan’ dormant buds after dehydration ranged from 0.47 to 0.70 gH_2_O g^-1^DM and from 0.29 to 0.66 gH_2_O g^-1^DM, respectively. The dehydration time of the D variant ranged from 10 to 14 days. The course of survival of ‘Sampion’ and ‘Spartan’ dormant buds during their cryopreservation sampled in the winter period of 1998/1999 is shown in **Figure 4**.

#### Winter Season of 1999/2000

The survival of ‘Sampion’ and ‘Spartan’ ND-30 variant ranged from 4 to 98% (December 29, 2000) and from 0 to 42%, respectively. The survival of ‘Sampion’ and ‘Spartan’ NDLN variant ranged from 0 to 89% (October 19, 1998) and from 2 to 44%, respectively. Water content of ‘Sampion’ and ‘Spartan’ dormant buds ranged from 0.85 to 1.20 gH_2_O g^-1^DM and from 0.73 to 0.95 gH_2_O g^-1^DM, respectively.

**FIGURE 5:**
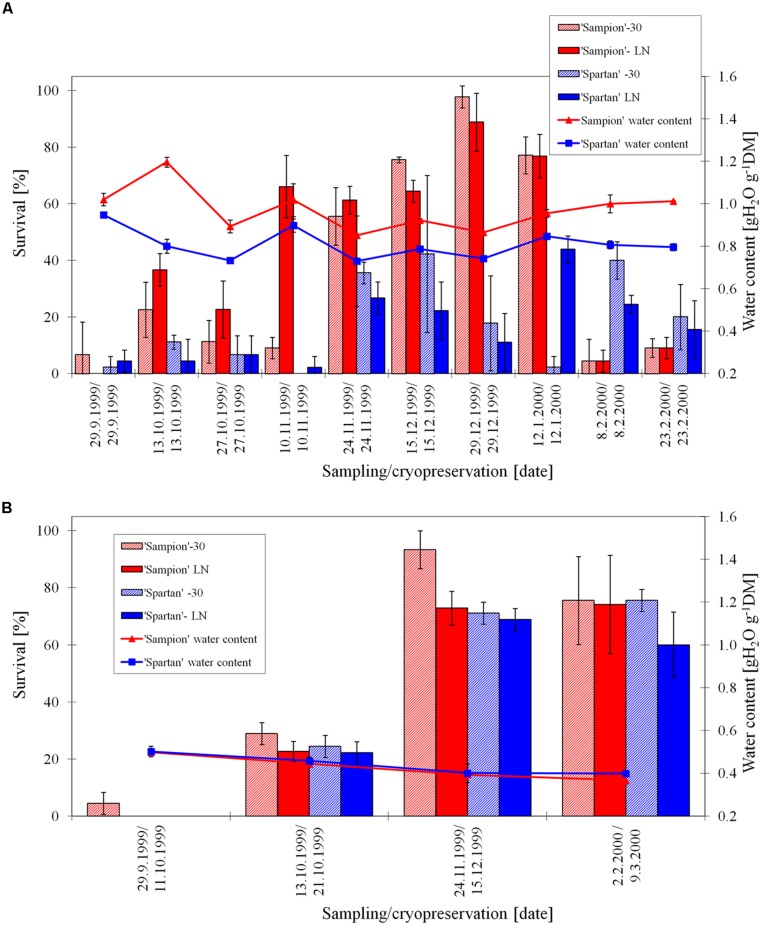
**Survival and water content of non-dehydrated **(A)** and dehydrated **(B)** vegetative buds of apple cultivars ‘Sampion’ and ‘Spartan’ **(B)** in the seasons of 1999/2000.** ND was cryopreserved immediately after sampling from the orchard. D variant was cryopreserved after frost dehydration of at least 1 week at –4°C. Survival was evaluated after the first step of cryopreservation protocol at –30°C (–30), and after the second step, reaching the LN temperature. Survival was tested by examination of oxidative browning of tissues on the longitudinal cut of the bud. Water content was measured immediately before cryopreservation procedure. Bars indicate SD (*p* < 0.05).

The survival of ‘Sampion’ and ‘Spartan’ D-30 variant ranged from 4 to 93% and from 0 to 76%, respectively. The survival of ‘Sampion’ and ‘Spartan’ DLN variant ranged from 0 to 74% and from 0 to 69%, respectively. Water content of ‘Sampion’ and ‘Spartan’ dormant buds after dehydration ranged from 0.37 to 0.50 gH_2_O g^-1^DM and from 0.40 to 0.50 gH_2_O g^-1^DM, respectively. The dehydration time of the D variant ranged from 8 to 36 days. The course of survival of ‘Sampion’ and ‘Spartan’ dormant buds during their cryopreservation sampled in the winter period of 1999/2000 is shown in **Figure 5**.

#### Winter Season of 2000/2001

The survival of ‘Sampion’ and ‘Spartan’ ND-30 variant ranged from 29 to 100% and from 0 to 93%, respectively. The survival of ‘Sampion’ and ‘Spartan’ NDLN variant ranged from 0 to 100% and from 0 to 42%, respectively. Water content of ‘Sampion’ and ‘Spartan’ dormant buds ranged from 0.89 to 0.99 gH_2_O g^-1^ and from 0.81 to 0.88 gH_2_O g^-1^, respectively.

**FIGURE 6:**
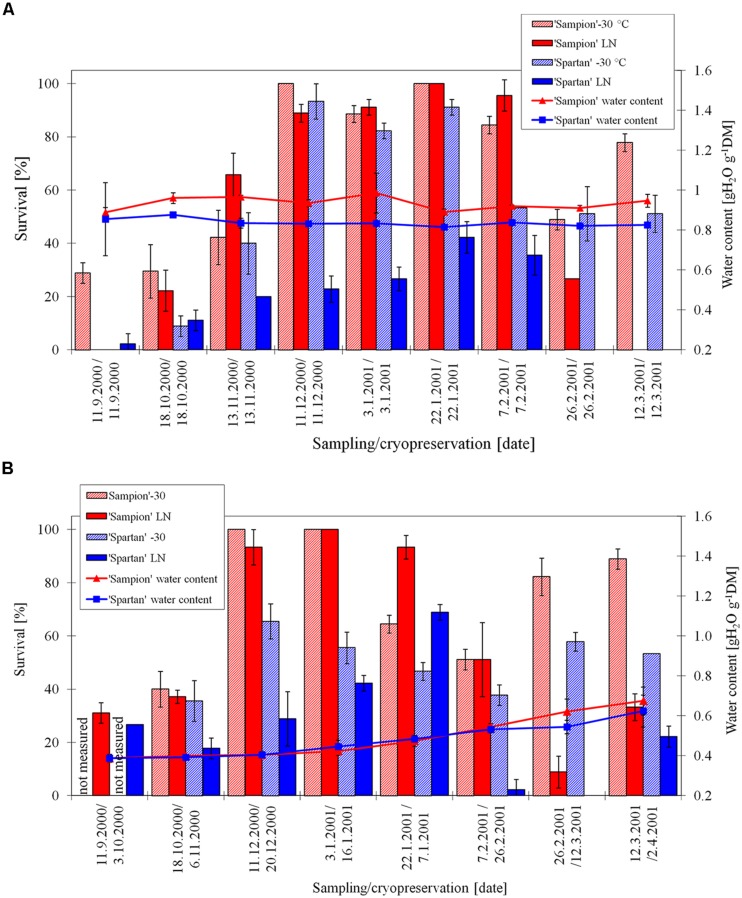
**Survival and water content of non-dehydrated (A) and dehydrated (B) vegetative buds of apple cultivars ‘Sampion’ and ‘Spartan’ **(B)** in the seasons of 2000/2001.** ND variant was cryopreserved immediately after sampling from the orchard. D variant was cryopreserved after frost dehydration of at least 1 week at –4°C. Survival was evaluated after the first step of cryopreservation protocol at –30°C (–30), and after the second step, reaching the LN temperature. Survival was tested by examination of oxidative browning of tissues on the longitudinal cut of the bud. Water content was measured immediately before cryopreservation procedure. Bars indicate SD (*p* < 0.05).

The survival of ‘Sampion’ and ‘Spartan’ D-30 variant ranged from 40 to 100% and from 36 to 66%, respectively. The survival of ‘Sampion’ and ‘Spartan’ DLN variant ranged from 9 to 100% and from 0 to 69%, respectively. Water content of ‘Sampion’ and ‘Spartan’ dormant buds after dehydration ranged from 0.39 to 0.67 gH_2_O g^-1^ and from 0.39 to 0.62 gH_2_O g^-1^, respectively. The dehydration time of the D variant ranged from 9 to 22 days. The course of survival of ‘Sampion’ and ‘Spartan’ dormant buds during their cryopreservation sampled in the winter period of 2000/2001 is shown in **Figure 6**.

## Discussion

The period of endodormancy of vegetative buds of studied apple cultivars ‘Sampion’ and ‘Spartan’ considerably differed in rated seasons (**Figure 2**). The first season, 1998 had a usual sum of temperatures and precipitation, the second season, in 1999, was hot and dry, and the third season had a usual sum of temperatures but was dry in comparison to average temperature and precipitation. Photoperiod changes were not evaluated because they do not induce dormancy in apples (Nichols et al., 1974; Heide and Prestrud, 2005). The earlier entering of buds into endodormant phase correlated with higher temperatures and low precipitation in 1999/2000. Normal temperatures and low precipitation in 2000/2001 caused shift of endodormancy to mid winter and shortened the length of endodormancy contrary to the first season of 1998/1999.

The shift of endodormancy phase to later winter in 2000/2001 season, contrary to normal season of 1998/1999, may be due to higher temperatures. That fact is consistent with published data of low-temperature induction of endodormancy (Heide and Prestrud, 2005). Although total precipitation and draft period are important factors inducing growth cessation and subsequent growth restoration, the impact of the drought season on entering endodormancy is questionable (Borchert, 1991). Dreyer et al. (1986) found that extreme and short drought periods (several weeks) at walnut trees during summer stopped temporarily the growth. This ecodormant phase consequently modified following endodormancy compared with regularly watered trees. Moreover, the bud dormancy in November was deeper in buds from watered trees and shallower in buds from trees exposed to drought (Dreyer et al., 1986). These authors proposed that the changes in the endodormancy were caused by disturbances in shoot growth dynamics in droughted trees. Similarly in our study, the dry and hot season in 1999 (**Figure 1**) could cause the shift of beginning of the endodormant period to earlier summer time, and also the shortening of endodormancy within the end of November.

We expected the water content of dormant buds to decrease in the beginning or in the endodormant phase, according to Faust et al. (1991), but the water content was unstable or maintained at approximately the same level. On the other hand, Erez et al. (1998) found that water content in fruit tree buds correlated more with frost resistance than with the level of endodormancy. Our results showed that the fluctuation of bud water content was superimposed with precipitation (not showed). On average, the water content was higher of approximately 0.1 gH_2_O g^-1^DM at cultivar ‘Sampion’ than at cultivar ‘Spartan’.

Despite of the fact that both of the cultivars belong to cultivars with similar earliness and cold hardiness, the length of endodormancy is longer in cultivar ‘Spartan’ than in cultivar ‘Sampion’. Also in both seasons with known whole length of endodormancy, the cultivar ‘Spartan’ had later exit from endodormancy. Only the second season, 1999/2000, both of the cultivars had the exit from endodormancy at the same time. The reasons for such differences might be due to different genotypes, demands of different cold requirements, and different age of trees of both cultivars (Hauagge and Cummins, 1991a,b).

When assessing the effect of dormancy of apple buds on survival after cryopreservation it is necessary to take into account the phase and course of dormancy. The highest survival of apple buds in seasons of 1998/1999 (**Figure 4**) and 2000/2001 (**Figure 6**) was at the end of the endodormant period (**Figure 2**). In contrast, in the season of 1999/2000, where the entire course of dormancy was unknown (**Figure 2**), the highest survival after exposure to LN was measured in buds sampled during December and January, after their endodormant period, in ecodormancy (**Figure 5**).

**FIGURE 7:**
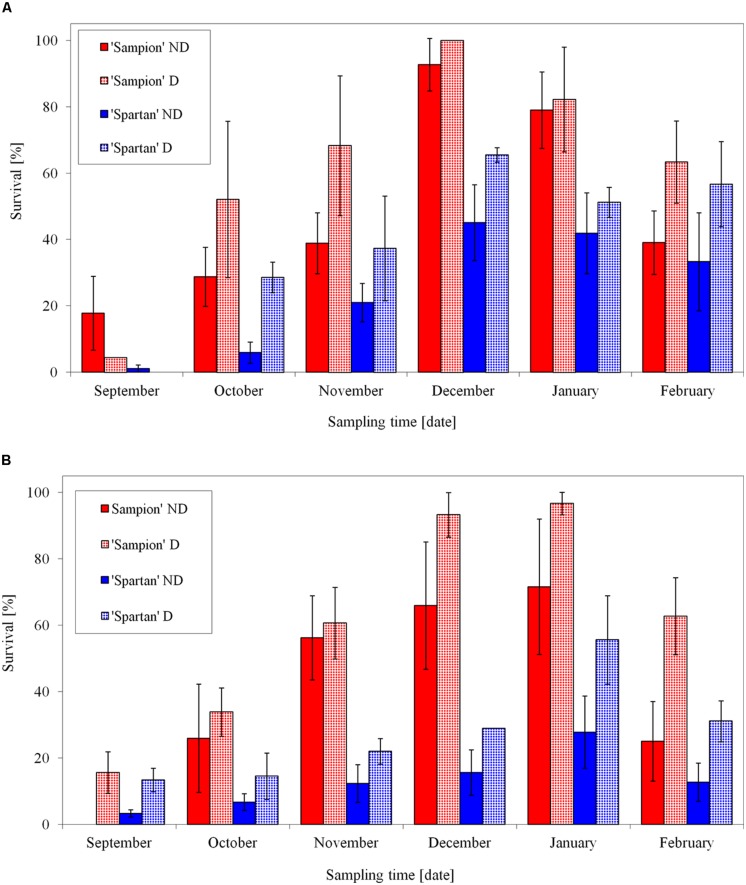
**Average survival from three seasons of 1998/1999, 1999/2000, and 2000/2001 of vegetative buds of apple cultivars ‘Sampion’ and ‘Spartan’ after the first step of cryopreservation protocol at –30°C **(A)**, and after the second step, reaching the LN temperature (B).** ND variant was cryopreserved immediately after sampling from the orchard. D variant was cryopreserved after frost dehydration of at least 1 week at –4°C. Survival was tested by examination of oxidative browning of tissues on the longitudinal cut of the bud. Water content was measured immediately before cryopreservation procedure. Bars indicate SD (*p* < 0.05).

After the first step of the cryopreservation (to –30°C) both cultivars had the highest survival in December contrary to the lowest bud survival in September (**Figure 7A**). The differences in survival were found either between individual apple cultivars or between ND and D variants in each cultivar. Dehydrated buds of both cultivars showed a higher frost survival after –30°C, after the first step of cryopreservation protocol. It was mainly in October and November, when the buds were not cold acclimated yet. The used dehydration procedure enhanced cold acclimation. The maximal survival after the first cooling to –30°C was prerequisite for the following second step of the cryopreservation protocol. Cold hardiness, acquired either early in the dormant season during good acclimation conditions (Stushnoff, 1991) or later in winter after reacclimation at artificial storage at –4°C (Forsline et al., 1998), is considered a key step for successful cryopreservation. This is in consistence with our results (**Figure 7**). Aronen and Ryynanen (2014) found in hybrid aspen that the time schedule for cryopreservation of dormant buds could be extended from mid-winter to late autumn without compromising the recovery of the cryostored material. From October to February, on average, over 75% of their cryopreserved buds could be regenerated by micropropagation.

After the second step of cryopreservation, the buds of both cultivars showed the highest survival in January (**Figure 7B**). While the cultivar ‘Sampion’ reached the highest average survival of 97%, ‘Spartan’ reached the highest average survival of 56% of the surviving buds (significantly different at α = 0.05). The differences in survival after cryopreservation among apple cultivars were found by several authors (Tyler et al., 1988; Forsline et al., 1998). Towill et al. (2004) evaluated several *Malus* germplasm accessions including different *Malus* species and they proposed that there is no strong relationship between variability after cryopreservation and phylogeny.

Survival of buds after the cryopreservation procedure was evaluated visually by examination of browning on the bud cut in our experiments. The examination of oxidative browning in tissues held in peat or 100% RH for several days was used as an estimate of viability/cold hardiness in a wide range of woody species (Towill and Bonnart, 2005). Although this evaluation was based on subjective estimation, it correlated with evaluation done by grafting of buds on rootstocks (*R*^2^ = 0.93) (Seufferheld et al., 1999). With the use of visual evaluation of bud cryosurvival and evaluation of shoot growth after grafting on rootstocks in orchard we obtained similar results of cryosurvival of more than 50 apple cultivars introduced in the Czech Cryobank with the average regrowth rate of 80% of grafted buds (unpublished results).

The buds in our study dehydrated less in some sampling dates, e.g., cultivar ‘Sampion’ from November 2, 1998; 0.62 gH_2_O g^-1^DM, (**Figure 4B**), sampling from later than February 7, 2001 cultivar ‘Sampion’; 0.70 gH_2_O g^-1^DM, (**Figure 6B**). The level of dehydration can be influenced by the size and length of the one-nodal segments, the smaller segments the faster dehydration (Forsline et al., 1998). Our findings were in contrary to their results, even though we used their recommendation for dehydration level of dormant buds to a moisture content of approximately 20–30% of moisture (0.25–0.43 gH_2_O g^-1^DM). In our experiments, the dehydration time was used according to Tyler and Stushnoff (1988b), who found the dehydration time of more than 10 days sufficient to induce a higher survival after LN exposure. Although, it is possible to cryopreserve buds of very cold hardy species, that naturally tolerate freezing to –30°C or below, using two-step cryopreservation procedure with slow freezing in the first step (Tyler et al., 1988; Forsline et al., 1998; Towill et al., 2004), a controlled dehydration treatment applied before cryopreservation procedure enhances viability, broadens spectrum of cultivars that can be cryopreserved, and extends the period for sampling plant material (Stushnoff, 1987; Tyler and Stushnoff, 1988a,b). On the other hand, dormant buds of some *Malus* species do not need desiccation prior to cooling to LN temperatures (Towill and Bonnart, 2005). This may be due to the differences in natural desiccation under natural winter conditions. In the *Malus* × *domestica* they found positive effect of dehydration; 95% of dehydrated accessions were successfully cryopreserved, contrary to 72% survival of non-dehydrated accessions.

Non-dehydrated buds, cryopreserved at the day of sampling from orchard, had water content from 0.64 to 1.20 gH_2_O g^-1^DM, compared to dehydrated buds ranged from 0.28 to 0.70 gH_2_O g^-1^DM. The non-dehydrated buds of cultivar ‘Sampion’ had higher amount of water content than cultivar ‘Spartan’ in almost all sampling dates (**Figures 4A, 5A**, and **6A**). Dehydrated buds of both cultivars had approximately similar water content (**Figures 5B and 6B**), only in the first season (**Figure 4B**), cultivar ‘Sampion’ had higher amount of water content than cultivar ‘Spartan’, which is similar to the situation in non-dehydrated buds. Surprisingly, the moisture content of the buds could only be lowered when the trees were dormant and well into cold acclimation without loss of bud viability after cryopreservation (Stushnoff, 1987).

Relationship between water content and survival of apple buds after cryopreservation was statistically significant only in cultivar ‘Sampion’ in 2000/2001 season. In other cases, there was not found positive effect of dehydration of apple buds on cryopreservation. To support our result, according to Seufferheld et al. (1999), we can speculate that in some dehydration procedures our buds could overcome the level to which they were acclimated at the time of sampling. However, Seufferheld et al. (1999) found buds collected just after the defoliation, but before cold acclimation, intolerant to dehydration. In addition, the buds collected in winters with warm period were less dehydration resistant (Stushnoff, 1987; Tyler and Stushnoff, 1988a,b). Although the water content had no statistically significant effect on bud cryopreservation survival, the positive tendency of lower water content is evident (**Figure 7**).

**FIGURE 8:**
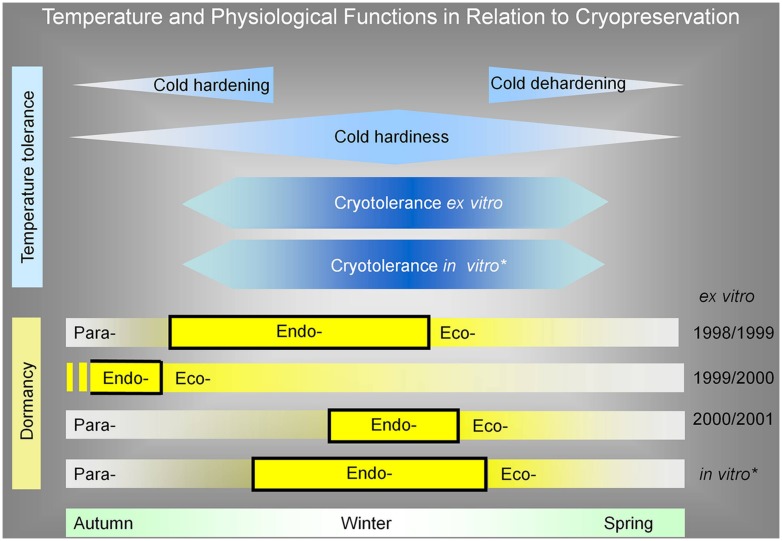
**Apple tree plant cryotolerance and cold hardiness as a reaction to low temperature in relation to its (para-, endo-, eco-) dormancy.** For comparison, the cryotolerance of *ex vitro* over all studied seasons was unified in time. The *in vitro* dormancy was added in the same range as *ex vitro* cryotolerance range. The dormancy and its parts were added in relation to evaluated cryotolerance range. From this expression, it is evident that the cryotolerance was not dependent on endodormancy, because, e.g., in the season of 1999/2000, the endormancy ended before the cryotolerace was established. The cryopreservation method for *ex vitro* dormant bud plants was two-step freezing, with pretreatment at –5°C for 2 months. The cryopreservation method for plants from *in vitro* was encapsulation/dehydration, without any pretreatment. The cryotolerance of *ex vitro* plants was determined after the immersion of buds in LN as a regrowth of new shoot from buds grafted on rootstock. The cryotolerance of *in vitro* plants was determined as new shoot regrowth in *in vitro* conditions. ^∗^Bilavcik et al. (2012).

One of the conclusions of our work is that cryotolerance of dormant apple buds is not directly dependent on endodormancy. On the other hand, in comparison to our published results of *in vitro* apple plant dormancy and cryotolerance (Bilavcik et al., 2012), the endodormancy of *in vitro* plants overlapped with the maximum of cold hardiness and cryotolerance (**Figure 8**). This might be caused by conducting *in vitro* experiments at controlled conditions; we did not reach the temperature and moisture disturbances of environmental conditions as in the natural conditions in the orchard. Although there was an idea to divide the influence of low temperature on cold hardiness and endodormancy, we were not able to set the experimental conditions. That is why these physiological factors were lined together as it was published in the literature dealing with cryopreservation.

Based on the results, it can be concluded that the effect of endodormancy on apple bud survival after the exposure to temperature of –196° C takes place only if closely connected with cold hardening. Because the period of endodormancy may occur during summer or early autumn period, it does not directly affect survival of buds after cryopreservation. On the other hand, the near-lethal stress can be responsible for releasing buds from endodormancy and thus it allows a successful regrowing of apple buds, otherwise being endodormant and did sprouting before cryopreservation (Wang and Faust, 1994). That consequently means that cryopreservation procedure attaining this stress may be necessary for successful sprouting of endodormant apple buds. Cox and Stushnoff (2001) found a comparable effect of exposure of buds of *Populus tremuloides* to LN causing bud break in normally endodormant buds. Although external factors affect onset and output of endodormancy, our results reveal that this dormant state, fortunately, does not limit the successful dormant apple bud cryopreservation. To the best of our knowledge, this is the first report of apple bud cryopreservation independency of the endodormancy state.

## Conflict of Interest Statement

The authors declare that the research was conducted in the absence of any commercial or financial relationships that could be construed as a potential conflict of interest.
